# About the Sterilization of Chitosan Hydrogel Nanoparticles

**DOI:** 10.1371/journal.pone.0168862

**Published:** 2016-12-21

**Authors:** Raquel Galante, Carolina F. Rediguieri, Irene Satiko Kikuchi, Pablo A. S. Vasquez, Rogério Colaço, Ana Paula Serro, Terezinha J. A. Pinto

**Affiliations:** 1 Departamento de Farmácia, Faculdade de Ciências Farmacêuticas—Universidade de São Paulo, Butantã, São Paulo, Brazil; 2 Centro de Química Estrutural, Instituto Superior Técnico—Universidade de Lisboa, Lisboa, Portugal; 3 Agência Nacional de Vigilância Sanitária, Brasília, Brazil; 4 Centro de Tecnologia das Radiações—Instituto de Pesquisas Energéticas e Nucleares, Butantã, São Paulo, Brazil; 5 Departamento de Engenharia Mecânica and IDMEC—Instituto Superior Técnico—Universidade de Lisboa, Lisboa, Portugal; 6 Centro de Investigação Interdisciplinar Egas Moniz, Instituto Superior de Ciências da Saúde Egas Moniz, Monte de Caparica, Caparica, Portugal; Helsingin Yliopisto, FINLAND

## Abstract

In the last years, nanostructured biomaterials have raised a great interest as platforms for delivery of drugs, genes, imaging agents and for tissue engineering applications. In particular, hydrogel nanoparticles (HNP) associate the distinctive features of hydrogels (high water uptake capacity, biocompatibility) with the advantages of being possible to tailor its physicochemical properties at nano-scale to increase solubility, immunocompatibility and cellular uptake. In order to be safe, HNP for biomedical applications, such as injectable or ophthalmic formulations, must be sterile. Literature is very scarce with respect to sterilization effects on nanostructured systems, and even more in what concerns HNP. This work aims to evaluate the effect and effectiveness of different sterilization methods on chitosan (CS) hydrogel nanoparticles. In addition to conventional methods (steam autoclave and gamma irradiation), a recent ozone-based method of sterilization was also tested. A model chitosan-tripolyphosphate (TPP) hydrogel nanoparticles (CS-HNP), with a broad spectrum of possible applications was produced and sterilized in the absence and in the presence of protective sugars (glucose and mannitol). Properties like size, zeta potential, absorbance, morphology, chemical structure and cytotoxicity were evaluated. It was found that the CS-HNP degrade by autoclaving and that sugars have no protective effect. Concerning gamma irradiation, the formation of agglomerates was observed, compromising the suspension stability. However, the nanoparticles resistance increases considerably in the presence of the sugars. Ozone sterilization did not lead to significant physical adverse effects, however, slight toxicity signs were observed, contrarily to gamma irradiation where no detectable changes on cells were found. Ozonation in the presence of sugars avoided cytotoxicity. Nevertheless, some chemical alterations were observed in the nanoparticles.

## Introduction

Medical device related infections are becoming an increasing prevalent area of infectious disease that contribute greatly to the increasing costs on health care systems [1]. The outbreak in the field of nanotechnology, the growing interest that nanomaterials have raised in pharmaceutical and nanomedicine fields and the high complexity that they may assume, triggered the need for developing strategies to guaranty the safety and effectiveness of these structures [2]. A nanosystem designed for biomedical applications (e.g. injectables, ophthalmic solutions) cannot be toxic or irritating and must be sterile [2].

Although aseptic manufacturing is often used for the production of sterile nanomedicines, it can be quite complicated, especially when multiple handling steps have to be done in sterile environments. Terminal sterilization is safer in biological terms, leading to a higher overall efficiency, and thus must be used whenever it is possible [3]. However, adverse effects may occur on the materials. Changes on the physicochemical characteristics of nanosystems can induce toxicity and loss of properties that lead to system failure [2, 4]. Few studies address the sterilization of nanostructured systems [2]. As far as the authors know, no work has been published regarding the sterilization of chitosan (CS) hydrogel nanoparticles (HNP).

HNP are a novel family of nanoscale materials with very promising characteristics to be used as platforms for the delivery of drugs, genes, imaging agents and for tissue engineering applications [5, 6]. These materials associate the distinctive features of hydrogels (high water uptake capacity, biocompatibility) with the advantages of being nanostructured, i.e. unique physicochemical properties attributed to their high surface area, shape and surface structure [6].

Chitosan presents interesting characteristics, e.g. biocompatibility, low toxicity, biodegradability, antimicrobial activity and low immunogenicity, which make it a promising material for several biomedical applications [7, 8]. In particular, chitosan nanostructures have been subject of a vast number of studies (e.g.[9, 10]) for this purpose. The cationic nature of chitosan allows the formation of stable ionic complexes with ions or polymers of opposite charge. In addition it has the ability to gel upon contact with special polyanions, a process referred as ‘ionotropic gelation’ [6, 11, 12]. This gelation process involves the formation of crosslinkings between/within polymer chains, mediated by the polyanions. One of the most widely used methods to obtain chitosan-based HNP is based on the ionotropic gelation of chitosan with sodium tripolyphosphate (TPP) [6, 11–17]. Although chitosan/TPP nanoparticles are still not commercially available, they have been the focus of several works, being reported as potential vehicles for the delivery of drugs to the ocular surface [9] and intravenous administration of drugs to treat neurological diseases [10]. According to United States Pharmacopeia (USP), both formulations are required to be sterile [18, 19].

The sterilization methods commonly used for polymers, including chitosan, comprise exposure to steam, ethylene oxide and gamma ray radiation [20–23]. These methods lead to changes in molecules of vital structures of the microorganisms. The current accepted sterility assurance level (SAL) is limited to 10^−6^ (no more than one viable microorganism in one million parts of final product is allowed [24]). Given the nature of action of the different forms of sterilization, they can also attack the materials by the same mechanisms, resulting in hydrolysis, oxidation, chain scission, and depolymerization [20–22, 25–27]. Ozone gas sterilization is a method with potential interest because of its low cost, it does not leave toxic residues and it can be applied to thermosensitive materials [28–32]. Although the mechanism of action of ozone is not yet fully understood, it is known that it oxidizes the constituents of the cell wall (e.g. proteins and amino acids), inducing lyses reactions and disruption of the cell membrane [33]. Its high oxidative power allows to eliminate virus, bacteria, protozoa and fungi in a quick and efficient way [33]. Nowadays, it is widely used in the sterilization of water and food [34–37], and also finds application in the sterilization of biomedical devices [28, 30, 32, 38, 39], since it is compatible with a large variety of biomaterials, such as stainless steel, titanium, ceramics, silicone-based materials and acrylates [40]. The information in the literature about the effect of ozone on hydrogels is very scarce. However good results were obtained with gelatin of animal origin [41].

The aim of this work was to study the influence of conventional and new sterilization approaches on the intrinsic properties of CS/TPP model nanostructured hydrogel (CS-HNP) in order to help in the selection of the more suitable method that minimizes undesirable effects on important properties of this type of nanostructured materials. Sterilization was carried out using gamma irradiation, steam autoclave and an innovative ozone treatment. The effects of sterilization were assessed through an extensive characterization of the materials, before and after sterilization. Properties like particles size, zeta potential, dispersion, chemical structure, morphology, and cytotoxicity were evaluated. Also, sterility tests were carried out to ensure the efficacy of the applied methods.

## Materials and Methods

### Materials

Low molecular weight medical grade (purified) CS (MW 296 kDa, deacetylation degree 82%) was kindly provided by Altakitin S.A; acetic acid (Glacial) 100% was purchased from Merck; TPP TG 85% and D-Manitol ≥98% from Sigma-Aldrich; sodium chloride (NaCl), sodium hydroxide (NaOH) and dextrose (all PA-ACS) from Synth^®^; tryptic soy broth (TSB) from Bacto^®^ and tryptic soy agar (TSA) from Difco^®^. *Geobacillus stearothermophilus* ATCC 7953 from 3M Attest was used for contamination purposes, due to its high resistance towards sterilization methods [30]. NCTC clone 929 (CCIAL 020) cell line of fibroblast from ATCC—CCL-1 was used for cytotoxicity assays, neutral red dye was acquired from National Aniline Division; Eagle medium from Sigma Aldrich.

### CS-HNP synthesis

CS-HNP were produced by ionic gelation method adapted from *Calvo et al*. [42], using TPP as ionic crosslinking agent. Preliminary studies with different concentrations of CS and TPP were conducted until a final proportion of CS:TPP ensured the formation of nanoparticles. Briefly, a solution of TPP 0.1% (in 0.9% NaCl) was prepared (pH 6). Also, a 0.1% CS solution was prepared by dissolving the polymer in 0.5% acetic acid in saline solution (0.45% NaCl) (pH 3.5). All preparations were filtered using a 0.45 μm pore size cellulose acetate filter. When necessary, pH was adjusted to required values with 1 M NaOH or 1 M HCl. The TPP solution was then added, drop wise, to the CS solution, under constant magnetic stirring (650 rpm), until the final proportion of 3:1 (v/v) (CS/TPP) was achieved (optimized proportion). The nanoparticles form spontaneously, creating an opalescent bluish suspension (final pH 4). After a setting period of 60 minutes, the suspension was divided in equal volume samples and kept in appropriate sealed containers at 4°C, until further use (typically no longer than one week, except for stability tests). In order to infer upon the possibility of a protective effect of sugars, such as mannitol or dextrose, an appropriate amount of these compounds was added to samples in order to achieve final sugar concentrations of 2.5 or 5%. Since the CS-HNP are pH-sensitive [43], when necessary, pH was readjusted to the initial value (pH 4) with 1 M NaOH or 1 M HCl, in order to avoid eventual changes in their physico-chemical properties. For Fourier transformed infrared spectroscopy (FTIR), scanning electronic microscopy (SEM) and transmission electronic microscopy (TEM) characterization, samples had to be dried (see respective sections). In these cases, they were previously dialyzed as described below, in order to eliminate the influence of the sugars and salt in the observations.

### CS-HNP characterization

#### Chemical structure

The chemical structure of the CS-HNP was studied by attenuated total reflection—Fourier transform infrared spectroscopy (ATR–FTIR)**.** The measurements were performed using a Perkin-Elmer FTIR spectrometer (Spectrum 1000). The spectra were recorded with a resolution of 4 cm^-1^ from 4000 to 500 cm^-1^. The CS-HNP suspensions containing glucose/mannitol were dialyzed (time: 18 h, dialysis medium: distilled and deionized water, membranes: tubing cellulose membranes with a molecular weight cut-off of 14,000 Da) and then freeze-dried (after 2 hours at -80°C, they were freeze-dried for 72 hours at a negative pressure of 100 microns and a temperature of -70°C) using a FTS System Dura-Dry MP.

#### Size and zeta potential

The particle size distribution of the CS-HNP suspension (1 mL) was measured using a Zetasizer equipment (Nano ZS, Malvern Instruments, UK, He–Ne laser λ = 633 nm, scattering angle 90°, at 25°C). The stability of the particles against agglomeration was evaluated by determination of the zeta potential values using disposable folded capillary zeta potential cells. All measured electrophoretic mobilities were converted into zeta potential using Smoluchowski’s formula. The Malvern Zetasizer software v7.10 was used for the analysis. In this study, values of viscosity (0.89 cP), refractive index (1.33) and dielectric constant (78.3) of water were used. Measurements were done at least in triplicate.

#### Conductivity and pH

The conductivity of the hydrogel nanoparticle suspensions before and after sterilization procedures was determined using a conductivity meter Cond 340i/SET from WTW, while the pH was measured with a bench-top pH meter inoLab^®^ pH 7110 also from WTW.

#### Morphology

Nanoparticle morphology was analyzed using a scanning electron microscope Hitachi S2400 15 KeV, and a transmission electron microscope Hitachi H-8100, 200 kV. For SEM analysis the samples were previously washed with dialysis membranes and then air-dried at room temperature or freeze-dried as referred in section 2.3.1. Prior to the SEM analysis the dried CS-HNP was covered by a 20 nm thin gold-palladium coating. For TEM analysis, the mesh grid was dipped in the nanoparticle suspension and then freeze-dried.

#### Absorbance

UV-Vis absorbance spectra of the nanoparticle suspension were obtained using a Thermo Scientific—Evolution 201 Spectrophotometer with a 1 nm resolution. Aliquots of 1 mL of the suspension were analyzed using saline solution as background and the data were recorded from 200 to 700 nm.

#### Cytotoxicity *in vitro*

The cytotoxicity assays were carried out by Agar Diffusion Testing following the procedures described in ISO 10993–5 and USP <87> [44]. Briefly, the cells of fibroblast L929 in monolayer was overlaid with agar and stained with a vital dye (neutral red). Samples were prepared as follows: sterile paper filter discs with a surface area of approximately 0.2 cm^2^ were immersed for 30 s in the hydrogel nanoparticle suspension. Latex fragments and non-toxic filter paper discs were used as positive and negative controls, respectively. The prepared samples were then placed in contact with the solidified agar layer (one per plate with 60 mm of diameter, containing 7 mL of cells suspension with a concentration of 3.5 x 10^5^ cells/mL), followed by a 24h incubation period, at 37 ± 1°C in a humidified incubator containing 5% of carbon dioxide. The plates were analyzed macroscopically and microscopically, and according to the zone extending from the samples, the biological reactivity was rated on a scale of grade N = 0 (no reactivity) to grade N = 4 (severe reactivity). All tests were performed in triplicate.

### CS-HNP sterilization

#### Gamma irradiation

The hydrogel nanoparticle suspensions were placed in closed test tubes and exposed to gamma radiation (cobalt-60) doses of 8, 13 and 25 kGy. The rate dose was between 5 and 6 kGy.h^-1^. During this procedure, the samples were kept at low temperature using ice pellets. All tests were carried out in triplicate. The radiation processing was performed at the Multipurpose Gamma Irradiation Facility at Instituto de Pesquisas Energéticas e Nucleares (IPEN).

#### Steam autoclaving

Samples were conditioned in closed glass tubes containing 5 mL of the hydrogel nanoparticle suspension. Steam sterilization was performed at 100, 110 and 121°C for 5, 10, 15 and 30 min in Autoclave Sercon equipment. All tests were carried out in triplicate.

#### Ozone gas

An ozone gas sterilization procedure already validated for medical devices (in accordance with ISO 14937) was used to sterilize the samples. A prototype sterilization chamber with 125 liter capacity, developed by Brasil Ozônio^®^ (São Paulo, Brazil) was used to expose samples to the ozone gas in a pulsed form [32]. Each pulse (P) was composed of four stages: vacuum, chamber filling, plateau (20 minutes), and vacuum. The humidity inside chamber was kept between 80–95%, with positive internal pressure ranging from 0.4 to 0.8 kgf/cm^2^ during chamber filling and negative internal pressure ranging from—0.8 to—0.4 kgf/cm^2^ during vacuum [32]. Ozone concentration, at the plateau stage, was kept between 35000–36000 ppm, and temperature process variations from 30–35°C. The 4 mL samples of the nanoparticle suspension were placed in cylindrical plastic test containers with diameter of 4 cm. In this case, the covers were not placed, so as to facilitate d≥iffusio≥ of gas. After completing the ozonation process, the caps were placed back on each sample with utmost care in order to avoid possible contamination. Samples were exposed to 2, 4, 8 or 10 pulses of ozonation (2P, 4P, 8P and 10P, respectively). All tests were carried out in triplicate.

#### Sterility tests

Sterility testes were carried out in order to certify the efficacy of the methods. The samples were submitted to a controlled contamination process with different amounts of bacterial suspension, to obtain different loads of spores (10^2^ 10^3^ and 10^4^ Colony Forming Units per milliliter, CFU/mL) from the biological indicator *Geobacillus stearothermophilus*. After sterilization, samples were transferred to test tubes containing tryptic soy broth (TSB) and then left to incubate for 14 days, at 56°C, period during which the turbidity was monitored to identify bacterial growth. All medium was prepared according to manufacture indications. In all assays, positive and negative controls were used (contaminated TSB and autoclaved TSB, respectively). All tests were carried out in triplicate.

### Statistical analysis

Statistical analyses were performed using R Project software v. 3.2.0. Measurements are presented as mean ± standard deviation, unless otherwise specified. ANOVA test was used to determine if the different group means are significantly different. Bonferroni’s test was done for multiple comparisons. When data do not follow a normal distribution (Shapiro Wilk test of normality was applied with α = 5%), Kruskal-Wallis Test was used to decide whether the population distributions were identical. The significance level chosen was α = 5%.

## Results and Discussion

### CS-HNP characterization before sterilization

It has been reported that several factors, such as pH, molecular weight of chitosan and its deacetylation level, may affect the physical and chemical properties of nanoparticles, and, in last instance, influence their stability [17, 45, 46]. Furthermore, as referred above, sterilization procedures may originate changes in the intrinsic properties of these materials. Thus, characterization of the physical and chemical properties of the CS-HNP was carried out before any sterilization, as starting point.

**Fig 1:**
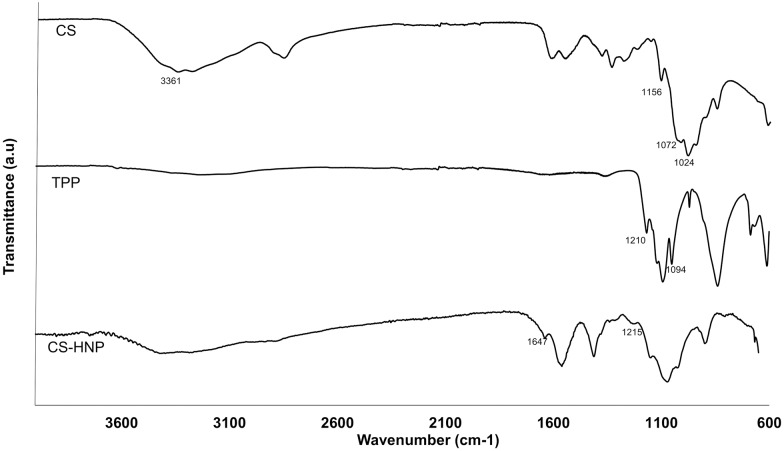
ATR-FTIR. Spectra data (CS, TPP and CS-HNP).

The formation of nanoparticles was assessed by FTIR analysis (Fig 1). It was possible to observe the main characteristic bands of pure CS, TPP and CS-HNP: for CS, the intense band at 3361 cm^-1^ (corresponding to stretching vibration of O-H and/or N-H), the absorption bond in 1156 cm^-1^ (C–O–C), in 1072 and 1024 cm^-1^ (vibrations concerning the C–O stretch) [17]; for TPP, the presence of the P-O and P = O groups at 1210 cm^−1^and 1094 cm^−1^, respectively. The formation of CS-HNP can be confirmed by the appearance of the absorption bands of the P-O groups (1215 cm^-1^) and of N-H deformations vibrations (1647 cm^-1^), which in turn are indicative of interaction between CS and TPP [17].

Size, polydispersity index (PDI), zeta potential, conductivity and pH were determined immediately after production and after 7 months of storage. Table 1 shows little or no variations in the studied properties. The nanoparticles average size increased with storage time suggesting a low degree of aggregation. PDI, a dimensionless parameter used to describe the variation in size, was calculated using the ZetaSizer equipment, according to ISO standard document 13321:1996 E and ISO 22412:2008. It is scaled from 0 to 1, in such as low values (<0.3) correspond to homogeneous suspension. Although it was observed a slight increase of the PDI value with the storage time, the CS-HNP suspension remained homogeneous (PDI < 0.3), which is desirable for pharmaceutical applications. Concerning zeta potential, it is indicative of the presence of repulsive forces and is widely used to predict the long-term stability of the particles. Nanoparticles tend to agglomerate to minimize the surface energy, as consequence of their high surface area [47]. The high values of zeta potential (around 25 mV) suggests that particles shall repel each other instead of coming together, which contributes to a stable suspension [47]. Commonly, for cationic nanoparticles (such as chitosan), values above 30 mV are associated with stable suspensions [47]. However, polymeric nanoparticles can be stabilized by esterification due to the influence of the chitosan chains molecular weight [47]. Conductivity and pH remained constant with storage time.

**Table 1:** CS-HNP physico-chemical characteristics over time.

	Size (nm)	PDI (a.u)	Zeta Potential (mV)	Conductivity (mS)	pH
After production	288±15	0.13±0.02	23.5±1.6	10.5±0.3	3.9±0.1
7 months storage (4°C)	314±2	0.19±0.03	25.1±0.1	10.7±0.5	3.9±0.1

**Fig 2:**
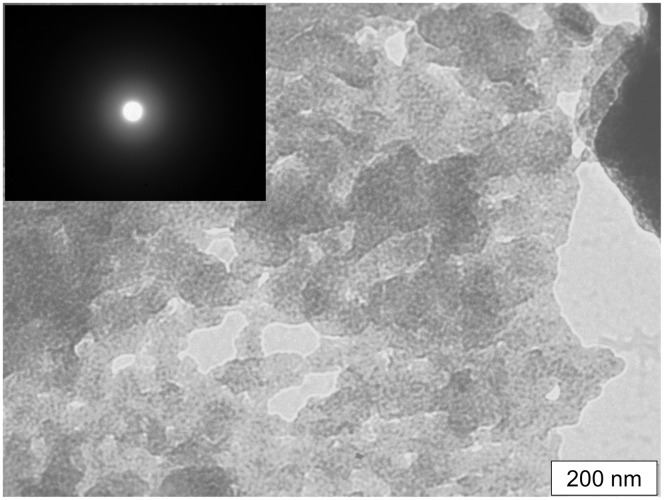
TEM. Observation of the untreated CS-HNP.

**Fig 3:**
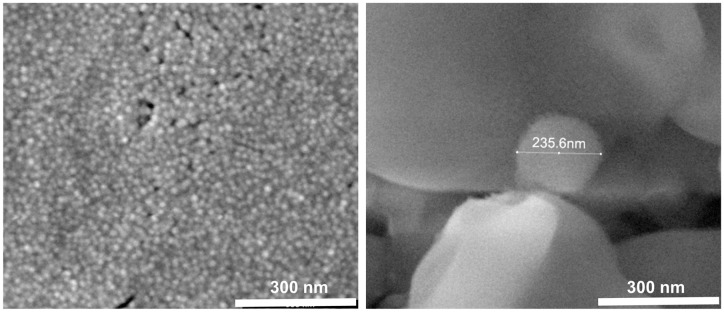
SEM. Images of the CS-HNP: A- air-dried (150 000x) and B- freeze-dried (150 000x).

The morphology of the CS-HNP was analyzed by TEM and SEM. TEM observations (Fig 2) made on a CS-HNP sample corresponding to untreated samples, showed particles with irregular rounded surface shape, whose dimensions of the longer axis are between 100–200 nm. The diffraction spot (inset on the upper left corner of the figure) showed that CS-HNPs, strongly agglomerated, are fully amorphous with no traces of crystallinity. SEM observations indicated that the size of the particles may change depending on the drying protocol that was used: air-dried CS-HNP 20–80 nm (Fig 3A), freeze-dried CS-HNP 200–300 nm (Fig 3B). This difference has been reported in other studies [17] and attributed to the fact that freeze drying involves sublimation of the water existent in the nanoparticles structure.

### Steam autoclaving

**Fig 4:**
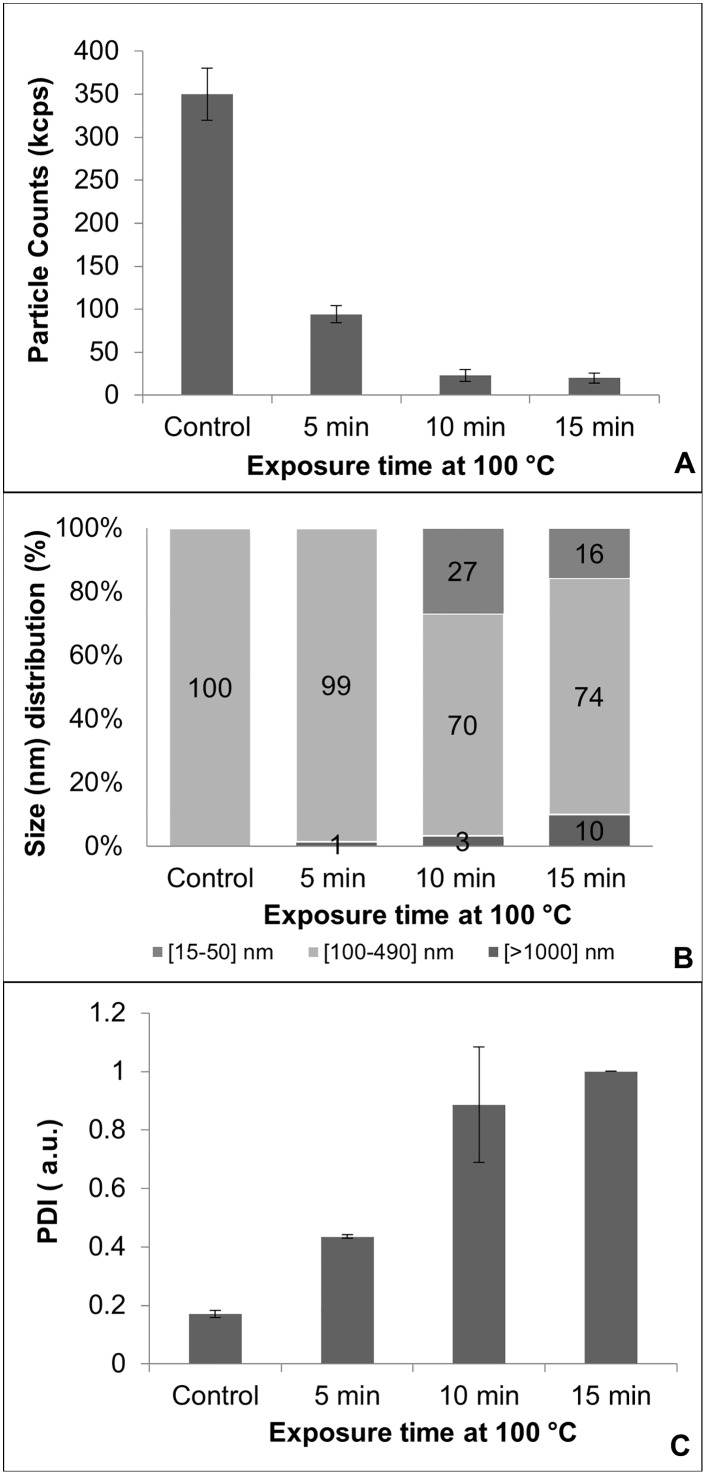
Zetasizer. Data for the autoclaved (100°C) CS-HNP suspensions: A- Particle counts, B- Average size distribution, C- PDI. Error bars correspond to standard deviations. (n ≥ 3).

CS-HNP suspensions were autoclaved at different temperatures (121, 110 and 100°C) and for different times (5, 10, 15 and 30 min). In all cases, signs of degradation were evident since the autoclaved samples became clear, losing their characteristic opalescent color. Zetasizer measurements of autoclaved samples revealed a poor quality suspension, with a very low particle count rate per second (<90kcps) when compared to the non-sterilized control samples, (>300 kcps), which is indicative of the severe alteration (Fig 4A).

The results showed very polydispersed samples, with several particle populations of different sizes, and presence of large aggregates and sediments (see Fig 4B and 4C for 100°C). The addition of sugars to the CS-HNP suspension, both glucose and mannitol, had no protective effect. Due to the poor quality of the autoclaved samples, no further characterization was pursued at this point, concerning this sterilization method.

### Gamma irradiation

**Fig 5:**
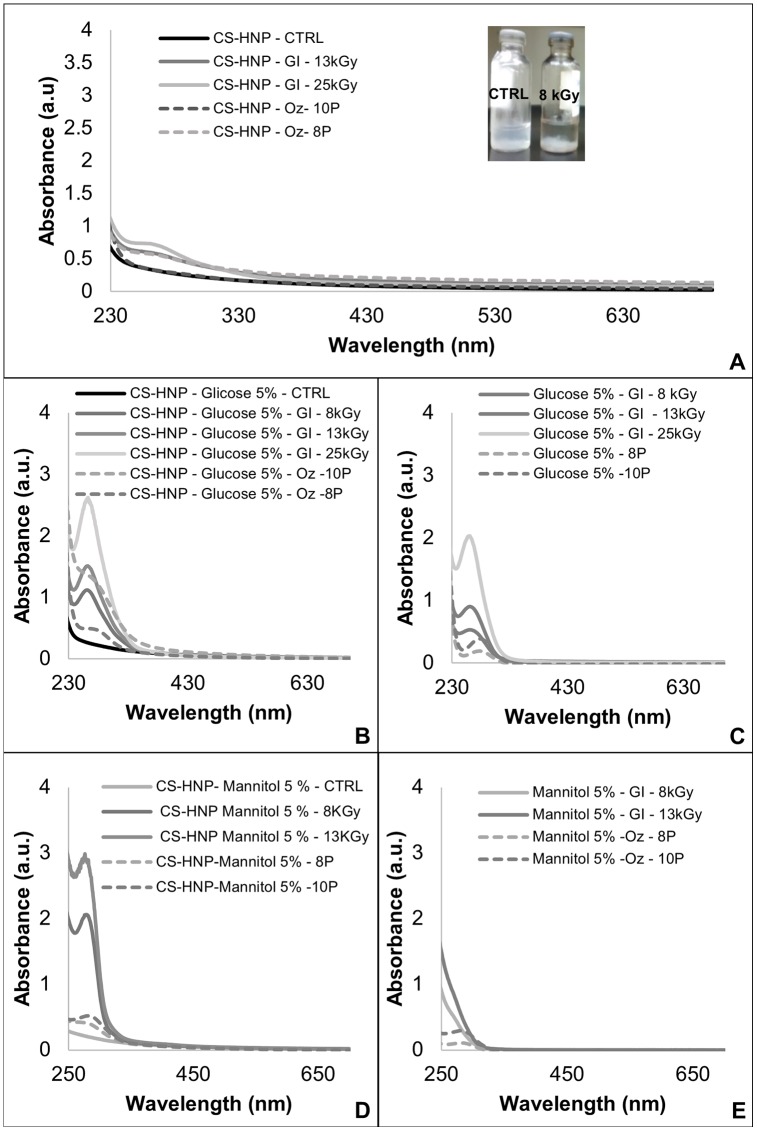
UV-Vis. Spectra of: A) Irradiated CS-HNP sample—the inset shows the sediment formation after irradiation, B) Irradiated CS-HNP in presence of Glucose 5%, C) Irradiated Glucose 5% solutions, D) CS-HNP in presence of Mannitol 5%, E) Irradiated Mannitol 5% solutions.

Exposing the CS-HNP suspension, as it is, to gamma rays, led to the immediate formation of visible sediments, even at the lower doses (Fig 5A).

**Fig 6:**
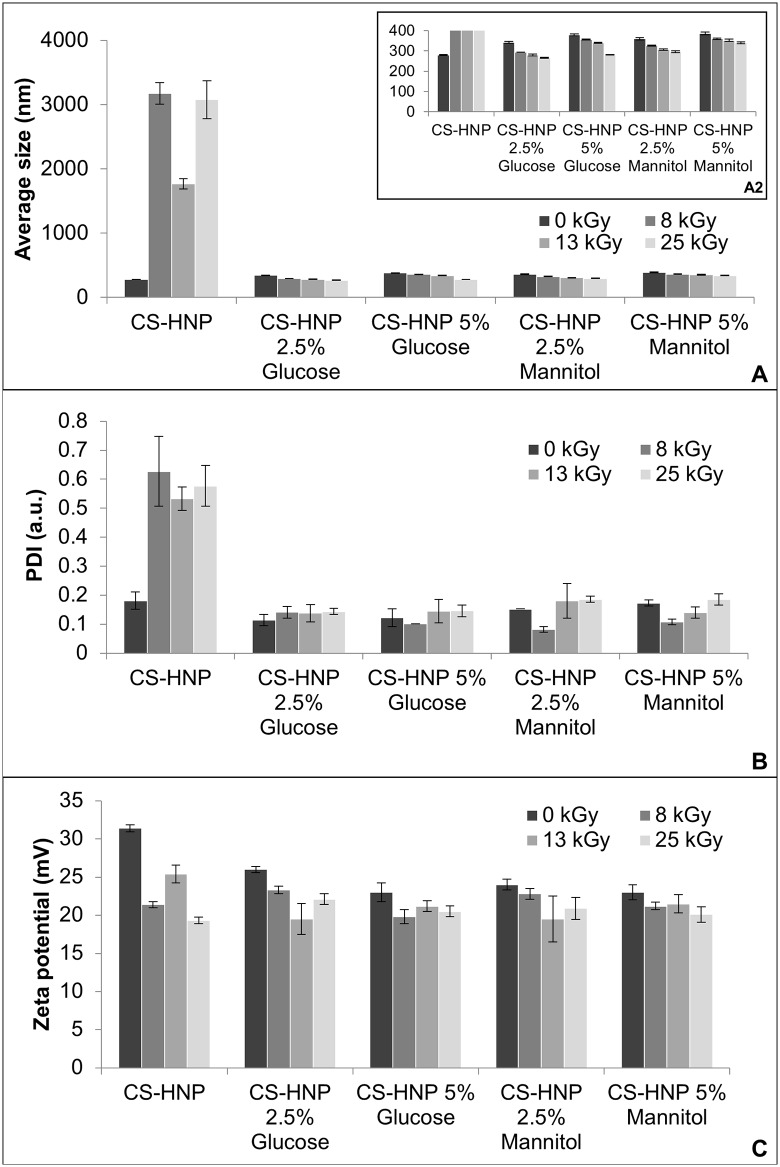
Zetasizer GI. Data for the irradiated CS-HNP suspensions: A- Average particle size, B- PDI and C- Zeta potential. Error bars correspond to standard deviations.

Although the UV-Vis spectra data did not show significant alterations (Fig 5A), the Zetasizer measurements showed considerable effects on the average particle size, PDI and zeta potential values, as displayed in (Fig 6), suggesting severe degradation of the CS-HNP. The average size of the particles significantly increased (*p-value*<0.001) as well as the polidispersivity (*p-value*<0.001), and the zeta potential decreased comparatively to control samples for all irradiation doses (*p-value*<0.001), which is in agreement with a higher propensity for agglomeration. Conductivity and pH values almost did not change relatively to the non-sterilized samples.

Different quantities of glucose and mannitol were added to the CS-HNP suspension, to infer upon the possible protective role of sugars. The obtained results expressed a significant increase of the nanoparticles resistance to radiation exposure in the presence of both mannitol and glucose. Size and PDI values variations relatively to the control samples were significantly lower after irradiation (Fig 6A and 6B). This demonstrated that the presence of sugars avoided the formation of aggregates. After the sugars addition, a decrease in particle size with the increase of the radiation dose was observed (*p-value*<0.001) (Fig 6A inset). With regard to the zeta potential parameter a tendency to decrease was observed after irradiation (Fig 6C), which was lower in the presence of sugars. However, taking into account the stabilization mechanism by esterification previously referred, suspensions with 20 mV may also be acceptable [47].

For glucose containing irradiated samples, the UV-Vis spectral data (Fig 5B) revealed the onset of a distinctive peak around 267 nm. This peak shall have two distinctive contributions associated: alterations suffered by the sugar, as can be proven from the UV-Vis spectral data of irradiated glucose solutions, where the same behavior was observed at the same wavelength (Fig 5C), and formation by action of the radiation of a chitosan-glucose complex between the chitosan and excess glucose. This complex has been reported as a high antioxidant substance [48, 49], which can be responsible for the protective effect against radiation, namely the nanoparticles’ no aggregation. For mannitol containing irradiated CS-HNP samples (Fig 5D), the UV-Vis spectra revealed a peak around 280 nm that was not observed in the irradiated mannitol solutions. However, the mannitol solutions (without CS-HNP) presented an increase of absorbance with the increase of the radiation dose (Fig 5E). These facts could indicate that both the CS-HNP and mannitol suffered some kind of alterations. The same tendencies were observed for the lower concentration sugar (2.5%) both with glucose and mannitol. Gamma irradiation produces as primary species H^·^ and OH^·^ radicals and solvated electrons, which induce chemical reactions with glucose and mannitol. The formed products shall be responsible for the shifts observed in the UV-VIS spectra. The identification of these products was not attempted in this work. According to H. Heusinger [50], one of the main products resulting from glucose radiolysis shall be malondialdehyde. Using gas-chromatography coupled to mass spectrometry this author also identified other fragmentation products, e.g. D-glyceraldehyde, D-glyceric acid, hydroxy-malondialdehyde, 2,3-dihydroxy-4-oxo-butanoic acid, tetrodialdose, D-arabinoic acid, D-xylose, 2,3,4-Trihydroxy-5-oxo-pentoic acid and D-arabinose. Concerning mannitol, the exposition to gamma irradiation leads to the oxidation of the primary alcohol groups in to aldehyde functions, producing D-mannose, followed by the secondary formation of arabinose [51].

**Fig 7:**
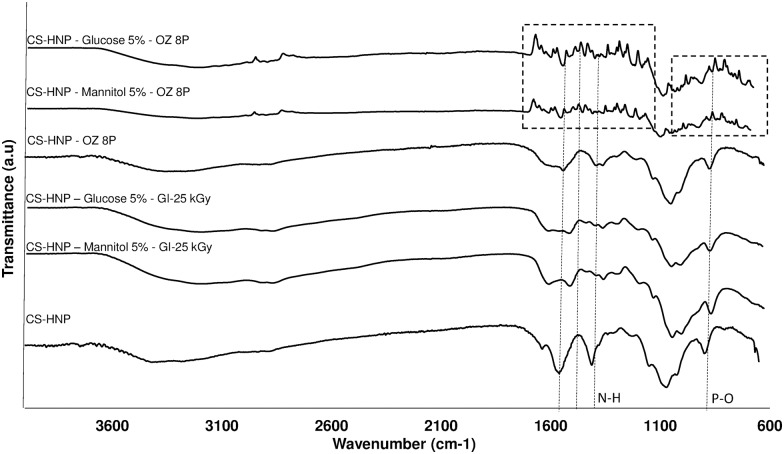
ATR-FTIR of sterilized samples. Spectra before and after CS-HNP sterilization.

From the ATR-FTIR data analysis (Fig 7), it was possible to conclude that even in the presence of sugars there were still some chemical alterations upon irradiation. The amides and PO groups seem to be the most affected by gamma rays (in both glucose and mannitol containing samples). It was not possible to identify significant changes between SEM images of the irradiated and non-irradiated.

Regarding bacteriologic safety, no bacterial growth was observed in the irradiated samples, in all tested conditions and for all loads of microorganisms (Table 2). Furthermore, for all analyzed irradiated samples no cytotoxic reactions were observed (N = 0 according to USP <87>) [44].

**Table 2:** Sterility and cytotoxicity tests results.

	Sterility			Cytotoxicity
Sample	10^2^ CFU/mL	10^3^ CFU/mL	10^4^ CFU/mL	N
Positive Control	Bg (day 1)	Bg (day 1)	Bg (day 1)	3
Negative Control	Sterile	Sterile	Sterile	0
Ozone 2 Pulses	Bg (day 1)	Bg (day 1)	Bg (day 1)	[N/A]
Ozone 4 Pulses	Bg (day 1)	Bg (day 2)	Bg (day 1)	[N/A]
Ozone 8 Pulses	Sterile	Sterile	Bg (day 1)	1
Ozone 10 Pulses	Sterile	Sterile	Sterile	1
Ozone 8 Pulses (Glucose/Mannitol)	Sterile	Sterile	Bg (day 1)	0
Ozone 10 Pulses (Glucose/Mannitol)	Sterile	Sterile	Sterile	0
γ Irradiation (Glucose/Mannitol) 8 kGy	Sterile	Sterile	Sterile	0
γ Irradiation (Glucose/Mannitol) 13 kGy	Sterile	Sterile	Sterile	0
γ Irradiation (Glucose/Mannitol) 25 kGy	Sterile	Sterile	Sterile	0

^**Sterile**: No bacterial growth for 14 days; **Bg (day X)**: Bacterial growth on day X; **N/A**: Not tested“; (N = 0): No reactivity, (N = 1): Slight reactivity (N = 2): Mild reactivity, (N = 3) Moderate reactivity, (N = 4): Severe reactivity”^

### Ozone gas

**Fig 8:**
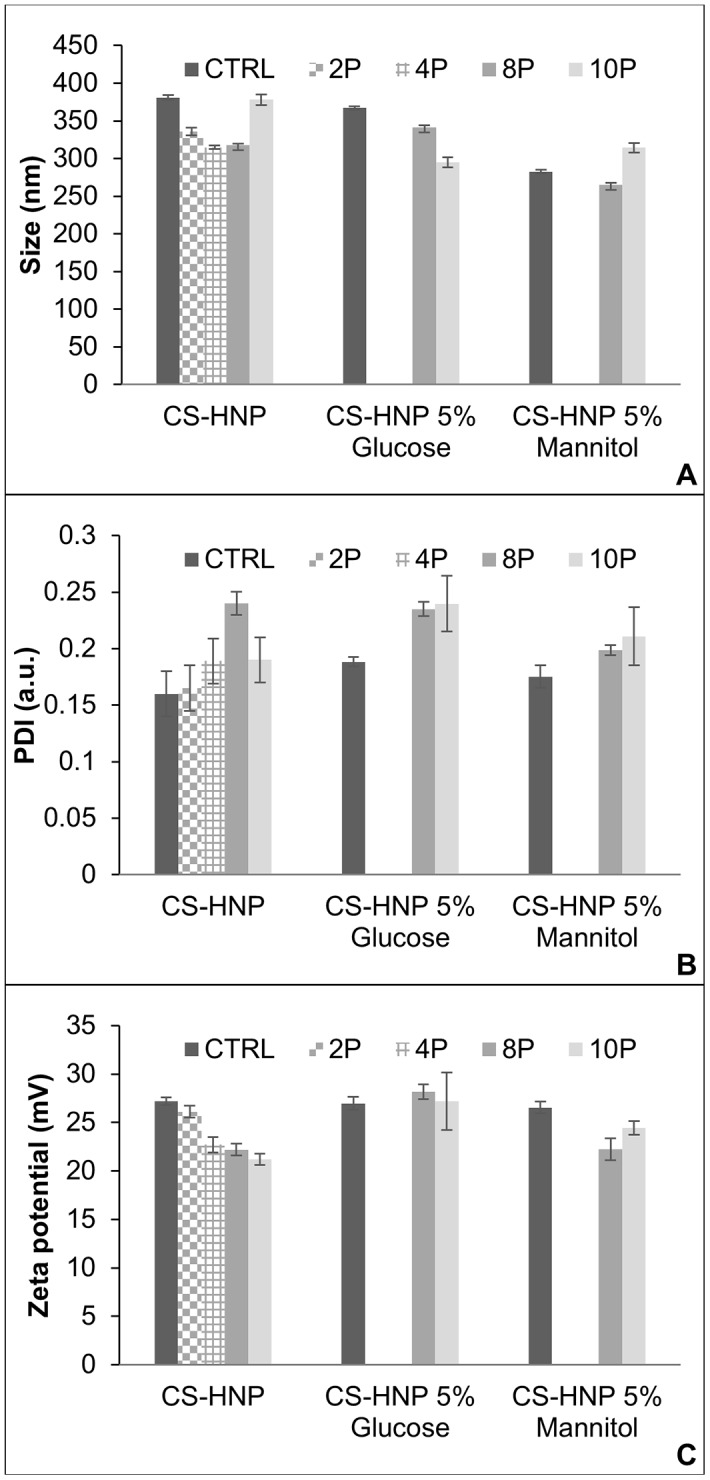
Zetasizer data for the ozonated CS-HNP suspensions. A- Average particle size B- PDI, C- Zeta potential. Samples containing glucose and mannitol were only exposed to 8P and 10P. Error bars correspond to standard deviations.

After the ozonation process, the nanoparticles average size presented a slight tendency to decrease relatively to the control (except for 10P) (Fig 8A). PDI values increased after treatment, however, this only was statistically significant for 8P (*p-value =* 0.0025), where it reached a value ~50% higher than the control (Fig 8B). Nevertheless, all PDI values remained below 0.3. Zeta potential decreased with the increase in the number of ozone pulses (Fig 8C), although remained above 20 mV.

Conductivity and pH values almost did not change relatively to the non-sterilized samples. The ATR-FTIR analysis did not show formation of new chemical groups nor significant alterations in the pre-existing peaks. The UV-Vis spectral data did not show alterations relatively to the control samples (Fig 5A). Furthermore, it was not possible to identify significant changes in morphology as a result from ozone exposure.

As for the efficacy, decontamination of 10^2^ and 10^3^ loads was only possible after 8 ozonation pulses, and no bacterial growth was observed in all samples when 10 ozonation pulses were applied (Table 2). However, in both cases (8 and 10P) some malformed or degenerated cells under specimens were observed, which corresponds to slight reactivity (N = 1) according to USP<87>.

Since 2 and 4P were not effective to sterilize the samples, experiments with the addition of sugar were only conducted for 8 and 10 P.

For glucose containing CS-HNP, a decrease in particle size (Fig 8A) was observed for 8P (*p-value =* 0.0026) and 10P (*p-value = 7*.*6e-06*). The PDI average value (Fig 8B) increased about 25% comparatively to the control, although it remained below 0.3. No significant changes were observed regarding zeta potential (Fig 8C).

Concerning mannitol containing samples, the nanoparticles presented a decrease in size for 8P (*p-value* = 0.014) and an increase for 10P (*p-value* = 0.00078), similar to the behavior observed for the samples without sugar. Changes in PDI relatively to the non-sterilized samples were not statistically significant. For zeta potential, a slight decrease was observed only for 8P (*p-value* = 0.0065).

The ATR-FTIR analysis of both glucose and mannitol containing CS-HNP showed that several new chemical groups are formed upon ozonation for 8P (Fig 7), indicating that the sugars interact chemically with the CS-HNP upon the ozonation process. However, this interaction did not lead to adverse biologic reactions, since no cytotoxic effects were observed (Table 2). The sterilization efficacy, when compared to the control samples, was not affected by the addition of sugars. Similar conclusions were achieved for 10P concerning the ATR-FTIR analysis, since spectra (not shown) were identical to those obtained for 8P. Also in that case (10P with addition of sugars) no cytotoxic effects were found and the sterilization efficacy remained equal to the one observed in the absence of sugars (Table 2).

In order to sterilize, ozone gas must diffuse through the aqueous phase of the nanoparticles suspension. When dissolved in water it can act in two different ways: either directly, or indirectly, through the formation of hydroxyl free radicals from water molecules [52, 53]. In acidic solutions, as our CS-HNP (pH 4), the direct action is predominant since there are more H^+^ ions available [54]. Since ozone diffuses better in air then in water, the aqueous phase hinders the sterilization efficacy. Therefore, the packaging conditions, as the geometry of the recipient (which determines the path of ozone diffusion in the aqueous medium), is critical. In this work, preliminary studies led us to choose a recipient geometry for the ozone treatment that minimizes the ozone path in the aqueous phase, as described previously.

## Conclusion

Sterilization is a crucial step in the production of materials for biomedical applications. For sensitive materials, such as CS-HNP, it is important to define procedures/conditions of sterilizations that do not damage the materials and allow the integrity of the desired properties to remain acceptable. In this work, the effect of several sterilization methods (steam heat, gamma irradiation and ozone) on the CS-HNP properties was evaluated.

The obtained results allowed concluding that steam heat is not a suitable method for sterilizing CS-HNP suspension as it leads to severe degradation of the samples, with the appearance of multiple particle populations of different sizes, and large aggregates and sediments. The addition of sugars did not demonstrate protective effect when steam heat sterilization was used.

Gamma rays exposure gave rise to immediate formation of visible sediments. However, upon the addition of protective sugars (glucose and mannitol 5%) a significant increase of the nanoparticles resistance to radiation was observed. This protective effect could be related with the formation of an antioxidant complex.

The ozonation process did not seem to affect the nanoparticles properties. However, it was not as effective as gamma irradiation and it originated signs of slight toxicity in the treated samples. The addition of sugars avoided the biologic reactivity without affecting the sterilization efficacy, nevertheless, chemical alterations in the FTIR spectral data were observed. For this method, in particular, the packaging conditions are especially critical, since they will determine the path of ozone diffusion through the natural barrier that aqueous medium constitutes.

## Supporting Information

S1 File ZetaSizer Data. Size, PDI and Zeta Potential data.
(XLSX)

S2 File Transmittance. UV-Vis spectral data.
(XLSX)

S3 File ZetaSizer Statistical. Size, PDI and Zeta Potential statistical data.
(XLSX)

S4 File Cytotoxicity. Cytotoxicity test data.
(PDF)
