# Supplementary material for: About the Sterilization of Chitosan Hydrogel Nanoparticles
Source: PLoS One. 2016 Dec 21;11(12):e0168862. doi: 10.1371/journal.pone.0168862 (PMC5176313; doi:10.1371/journal.pone.0168862)
Supplement: S4 File — Cytotoxicity test data. (PDF) [file pone.0168862.s004.pdf]

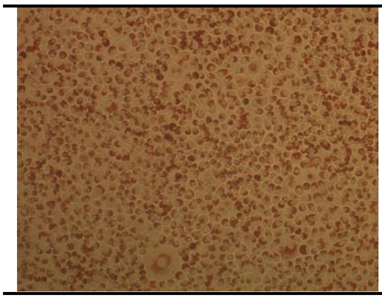

Negative Control

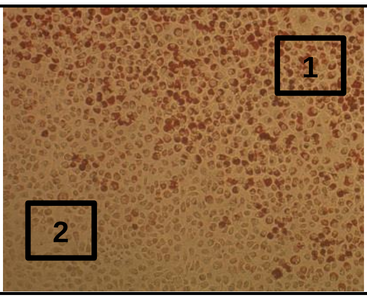

Positive Control

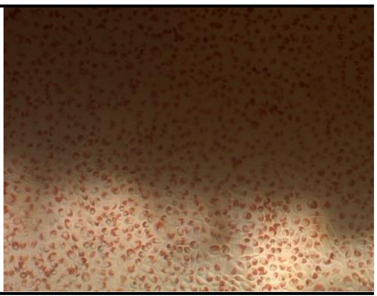

CS- HNP (**Filtration**)

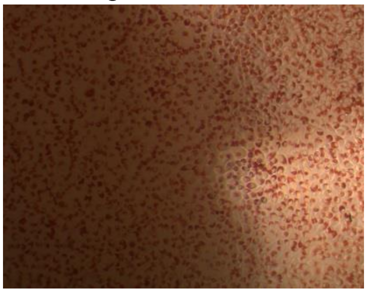

CS- HNP – Mannitol - 8 kGy

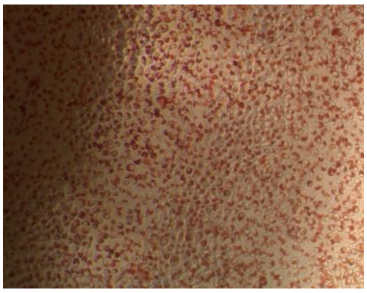

CS- HNP – Mannitol 13 kGy

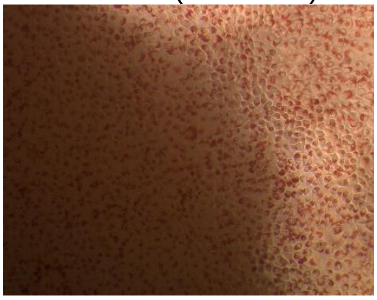

CS- HNP – Mannitol 25 kGy

1- Stained viable cells   2- Unstained dead cells

- Cell line from mouse connective tissue NCTC clone 929 (CCIAL 020) from ATCC - CCL-1. Maintained in Eagle's minimum medium supplemented with 0.1 mM nonessential amino acids, 1 mM sodium pyruvate and 10% fetal bovine serum without antibiotics.

- ATV - trypsin Association 0.20% and 0.02% versene mobile dispersion (peal of bottles).

- Petri plates 60 x 15 mm (Nunc Each plate was inoculated into 5 ml of the cell suspension at a concentration of 3 x 105 cells / ml

- Half Eagle's minimum serum 2X concentrated

- Agar (BD) at a concentration of 1.8%

- Neutral Red dye (National Aniline Division) at 0.01%

- Negative Control Filter paper J.Prolab

- Positive Latex Control - orthodontic elastic intraoral brand Morelli.

-
